# Utility of Pulse Oximetry Oxygen Saturation (SpO_2_) with Incorporation of Positive End-Expiratory Pressure (SpO_2_*∗*10/FiO_2_*∗*PEEP) for Classification and Prognostication of Patients with Acute Respiratory Distress Syndrome

**DOI:** 10.1155/2022/7871579

**Published:** 2022-09-06

**Authors:** Pratibha Todur, Anitha Nileshwar, Souvik Chaudhuri, Nitin Gupta, Srikant Natarajan, Shwethapriya Rao

**Affiliations:** ^1^Department of Respiratory Therapy, Manipal College of Health Professions, Manipal Academy of Higher Education, Manipal 576104, Karnataka, India; ^2^Department of Anaesthesiology, Kasturba Medical College, Manipal Academy of Higher Education, Manipal 576104, Karnataka, India; ^3^Department of Critical Care Medicine, Kasturba Medical College, Manipal Academy of Higher Education, Manipal 576104, Karnataka, India; ^4^Department of Infectious Diseases, Kasturba Medical College, Manipal Academy of Higher Education, Manipal 576104, Karnataka, India; ^5^Department of Oral Pathology & Microbiology, Manipal College of Dental Sciences, Mangalore 575001, Karnataka, India

## Abstract

**Background:**

Conventionally, PaO_2_/FiO_2_ (P/F ratio) has been used to categorize severity of acute respiratory distress syndrome (ARDS) and prognostication of outcome. Recent literature has shown that incorporation of positive end-expiratory pressure (PEEP) into the P/F ratio (PaO_2_*∗*10/FiO_2_*∗*PEEP or P/FP*∗*10) has a much better prognostic ability in ARDS as compared to P/F ratio. The aim of this study was to correlate SpO_2_*∗*10/FiO_2_*∗*PEEP (S/FP*∗*10) to PaO_2_*∗*10/FiO_2_*∗*PEEP (P/FP*∗*10) and evaluate the utility of S/FP*∗*10 as a reliable noninvasive indicator of oxygenation in ARDS to avoid repeated arterial blood sampling.

**Aim:**

To evaluate if pulse oximetry is a reliable indicator of oxygenation in ARDS patients by calculating SpO_2_*∗*10/FiO_2_*∗*PEEP (S/FP*∗*10). The primary objective was to determine the correlation of S/FP*∗*10 to P/FP*∗*10 ratio in ARDS patients. The secondary objective was to determine the cut-off value of S/FP*∗*10 ratio to predict severe ARDS and survival.

**Methods:**

Patients aged 18–80 years on invasive mechanical ventilation (MV) diagnosed with ARDS as defined by the Berlin definition were included. The values of PaO_2_, FiO_2_, and SpO_2_ were collected at three different time points. They were at baseline, i.e., after intubation and initiation of MV (within one hour of intubation), day one (1–24 hours of MV), and day three (48–72 hours of MV). The primary outcome was survival at the end of intensive care unit (ICU) stay.

**Results:**

A total of 85 patients with ARDS on invasive MV were included. The data points were obtained at baseline, day one, and day three of MV. S/FP*∗*10 ratio has an excellent correlation to P/FP*∗*10 ratio at baseline and day three of invasive MV (*r* = 0.831 and 0.853, respectively; *p* < 0.001) and has a strong correlation on day one of invasive MV (r = 0.733, *p* < 0.001). S/FP*∗*10 ratio ≤116 at baseline has excellent discriminant function to be categorized as severe ARDS as per Berlin definition (AUC: 0.925, *p* < 0.001, 90% sensitivity, 93% specificity, CI: [0.862–0.988]). The increase in S/FP*∗*10 ratio by ≥64.40 from baseline to day three of MV is a good predictor of survival (AUC: 0.877, *p* < 0.001, 73.5% sensitivity, 97% specificity, CI: [0.803–0.952]).

**Conclusion:**

S/FP*∗*10 has a strong correlation to P/FP*∗*10 in ARDS patients. S/FP*∗*10 ≤116 has an excellent discriminant function to be categorized as severe ARDS. The S/FP*∗*10 ratio on day three of MV and the change in S/FP*∗*10 ratio from baseline and day one to day three of MV are good predictors of survival in ARDS patients. This trial is registered with CTRI/2020/04/024940.

## 1. Introduction

The severity, oxygenation status, and extent of lung injury in acute respiratory distress syndrome (ARDS) have traditionally been assessed by the ratio of partial pressure of arterial oxygen to fraction of inspired oxygen (PaO_2_/FiO_2_ or P/F ratio) as per the Berlin definition [1]. However, the P/F ratio is often misleading in predicting the extent of lung injury [2, 3]. Two different mechanically ventilated patients with the same P/F ratio may have different positive end-expiratory pressures (PEEP). As the application of PEEP will improve the PaO_2_, calculating a P/F ratio without taking PEEP into account may be misleading [2]. A recent study has shown that PaO_2_*∗*10/FiO_2_*∗*PEEP (P/FP*∗*10 ratio) has a significantly better predictive ability for mortality in ARDS patients when compared to P/F ratio alone [2]. However, as these values change dynamically during the course of mechanical ventilation (MV) in a patient, repeated arterial sampling is required which is associated with increased chances of infection, blood loss, patient discomfort, and costs [4, 5].

## 2. Aim

The aim of this study is to evaluate oxygen saturation by pulse oximetry (SpO_2_) as an alternative noninvasive reliable indicator of oxygenation by calculating SpO_2_*∗*10/FiO_2_*∗*PEEP (S/FP*∗*10) ratio. SpO_2_ measurement is widely available, is conducive to use, enables continuous monitoring, and is reliable [6]. To the best of our knowledge, this is the first study that has incorporated the concept of PEEP into SpO_2_/FiO_2_ (S/F) to evaluate its accuracy in predicting severe ARDS and survival. The primary objective of the study was to determine the correlation of the S/FP*∗*10 ratio to P/FP*∗*10 in patients with ARDS. The secondary objective was to determine the cut-off value of the S/FP*∗*10 ratio to predict severe ARDS and survival.

## 3. Materials and Methods

### 3.1. Study Setting and Approvals

It was a single-centre prospective observational study conducted at the level III intensive care units (ICUs) of a tertiary care medical college from September 2020 to September 2021. This data was a secondary analysis of an ongoing prospective observational study approved by the Institutional Ethics Committee (IEC: 765/2019) and is registered in India's Clinical Trial Registry (CTRI/2020/04/024940).

### 3.2. Inclusion Criteria

The inclusion criteria were as follows: all patients between the ages of 18 and 80 years with ARDS, as defined by the Berlin definition, and on invasive mechanical ventilation.

### 3.3. Exclusion Criteria

The exclusion criteria were as follows:

- Patients with COVID-19.
- Patients with documented barotrauma (air leak syndromes).
- Patients with penetrating chest injuries.
- Patients planned for palliative care.

### 3.4. Sample Size

The sample size was based on the primary objective of assessment of correlation between two quantitative variables considering the expected correlation coefficient between PaO_2_*∗*10/FiO_2_*∗*PEEP (P/FP*∗*10) and SpO_2_*∗*10/FiO_2_*∗*PEEP (S/FP*∗*10) as at least 0.3, with power of 80% and alpha error of 5%. The following formula was used to calculate sample size (*n*):

(1) $$\begin{array}{c} n=\frac{{\left(Z_{1-\beta }+Z_{1-\left(\alpha /2\right)}\right)}^{2}}{\left(r^{2}/\left(1-r^{2}\right)\right)}, \end{array}$$

where *r* is the correlation coefficient, *Z*_1−*α*/2_ is the desired confidence level, and 1 − *β* is the power; thus, *n* = 85.

### 3.5. Methodology

All patients admitted to the ICU were screened daily for eligibility. Those who met the inclusion criteria were enrolled, after obtaining written informed consent from the legally authorized representatives.

**Figure 1:**
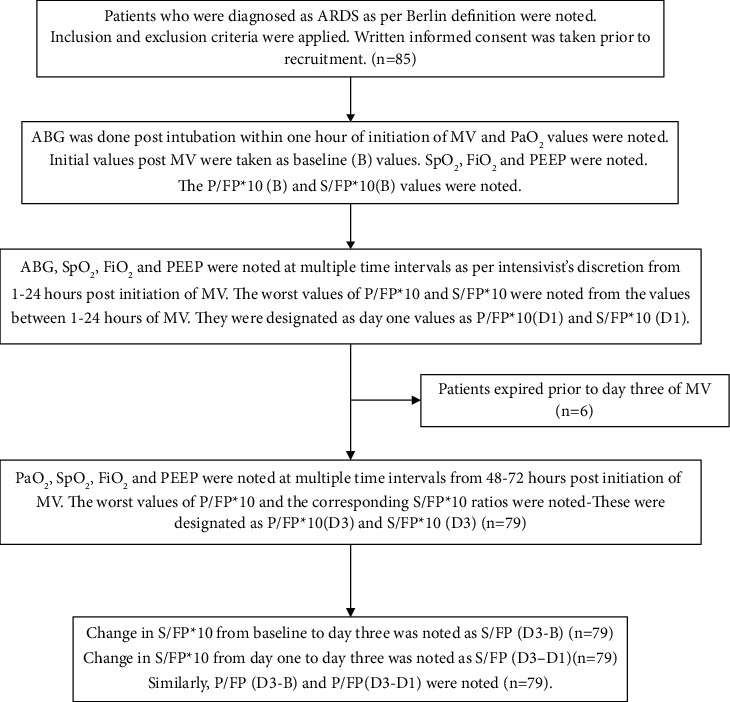
Flowchart depicting the methodology of the study.

The flowchart depicting the methodology is shown in Figure 1.

In Figure 1, ABG = arterial blood gas, MV = mechanical ventilation, PaO_2_ = partial pressure of arterial oxygen, SpO_2_ = pulse oximetry oxygen saturation, FiO_2_ = fraction of inspired oxygen, PEEP = positive end-expiratory pressure, P/FP*∗*10 = (PaO_2_*∗*10)/(FiO_2_*∗*PEEP), S/FP*∗*10 = (SpO_2_*∗*10)/(FiO_2_*∗*PEEP), P/FP*∗*10 (B) = the value obtained at one hour after intubation and initiation of MV, S/FP*∗*10 (B) = corresponding values obtained at one hour after intubation and MV, P/FP*∗*10 (D1) = worst values of P/FP*∗*10 day 1 (within 1–24 hours of intubation), S/FP*∗*10 (D1) = corresponding values of S/FP*∗*10 on day 1, P/FP*∗*10 (D3) = worst values of P/FP*∗*10 day 3 (within 48–72 hours of intubation), S/FP*∗*10 (D3) = corresponding values of S/FP*∗*10 on day 3 (within 48–72 hours of intubation), S/FP (D3–B) = change in S/FP*∗*10 from baseline to day three, S/FP (D3–D1) = change in S/FP*∗*10 from day one to day three, P/FP (D3-B) = change in P/FP*∗*10 from baseline to day three, and P/FP (D3-D1) = change in S/FP*∗*10 from day one to day three.

Sequential Organ Failure Assessment (SOFA) score on ICU admission was noted. PaO_2_ values were noted using the worst ABG during three defined time periods. The three time periods taken for the study were as follows: baseline (B: within one hour of intubation), day 1 (D1: 1–24 hours after intubation), and day 3 (D3: 48–72 hours after intubation). The FiO_2_, SpO_2_, and corresponding PEEP value set on the ventilator were also noted. The difference between day three and day S/FP∗10 values [S/FP (D3-D1] as well as the difference between the values of S/FP∗10 between that of day 3 and baseline [S/FP (D3-B)] were also calculated. Similarly, the difference between day three and day one P/FP∗10 values [P/FP (D3-D1] as well as the difference the values of P/FP∗10 between that of day three and baseline [P/FP (D3-B)] were also calculated. MV days, length of stay in ICU (ICU LOS), and outcomes in terms of survival at the end of ICU stay were also noted.

## 4. Statistical Analysis

The analysis of data was done using SPSS (Statistical Package for the Social Sciences) software (IBM Corp. Released 2012. IBM SPSS Statistics for Windows, version 22.0 Armonk, NY: IBM). Mean and standard deviation (SD) for the variables following parametric distribution were calculated. The analysis was performed for the three unique data points (baseline, day one, and day three of MV), and repeated data points were not aggregated for the purpose of analysis.

For correlation of the variables P/FP*∗*10 and S/FP*∗*10, the Pearson correlation test was used, and the correlation coefficient *r* was calculated. Pearson's correlation coefficient *r* <0.4 was considered as weak correlation, 0.4–0.59 was considered moderate correlation, *r* within 0.6–0.79 was considered as strong correlation, and *r* >0.8 was considered as excellent correlation. Additionally, the correlation analysis was also conducted for those patients with high PEEP (≥10 cm H_2_O). The main correlation analysis of S/FP*∗*10 and P/FP*∗*10 included patients at all PEEP levels (PEEP <10 cm H_2_O as well as PEEP ≥10 cm H_2_O). However, we separately did a sub-analysis of the correlation of S/F to P/F and S/FP*∗*10 to P/FP*∗*10 at the three time points. This separate sub-analysis was done because clinicians often repeat ABG for patients with higher PEEP levels, and thus it is this category of patients that is likely to benefit the most if the noninvasively determined S/FP*∗*10 correlated to P/FP*∗*10 at all the three time points.

A receiver operating characteristic (ROC) was plotted for the ability of the P/FP*∗*10 (B, D1, D3) and S/FP*∗*10 (B, D1, D3) to predict survival. Similarly, ROC curves were plotted for S/FP (D3-B), S/FP (D3-D1), P/FP (D3-B), and P/FP (D3-D1) to predict survival. Area under curve (AUC) within 0.91–1.0 was considered excellent, 0.81–0.90 was considered good, and 0.71–0.80 was considered fair. A *p* value <0.05 was taken as statistically significant and *p* value <0.001 as statistically highly significant.

To compare the reliability of S/FP to predict outcomes as compared to P/FP, the comparison of the ROC curves was done using the DeLong test using MedCalc software version 20.111 (Ostend, Belgium).

## 5. Results

A total of 85 patients with ARDS receiving invasive mechanical ventilation were included in the study. Six patients died between day one and day three. At the end of the ICU stay, 49 (58%) patients survived. The demographic characteristics and values of the variables of the 85 ARDS patients are depicted in Table 1.

**Figure 2:**
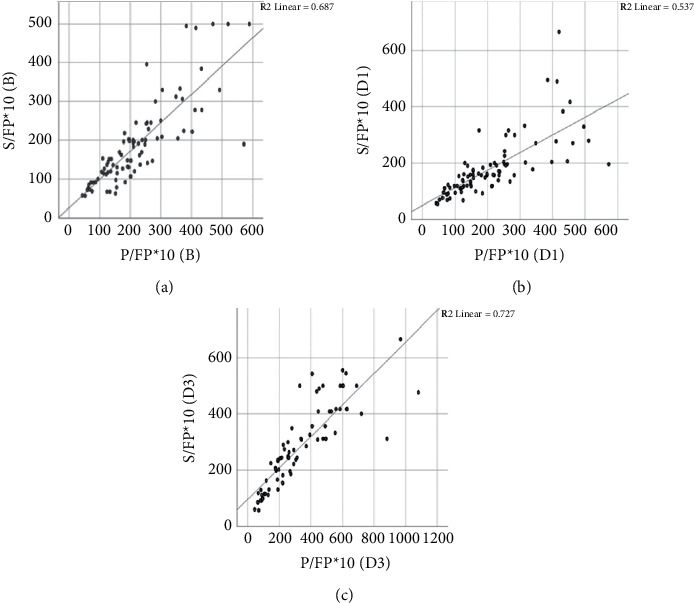
Scatterplot depicting correlation of S/FP*∗*10 and P/FP*∗*10 at all levels of PEEP at baseline (Panel (a)), day one (Panel (b)), and day three (Panel (c)). PEEP: positive end-expiratory pressure.

The correlation of P/FP*∗*10 and S/FP*∗*10 at baseline, day one, and day three of MV was stronger than the correlation of P/F and S/F on the respective days (Table 2). For the subgroup of patients with PEEP ≥10 cm H_2_O, the same correlation was observed as in all patients (Table 2). The scatterplot depicting correlation of S/FP*∗*10 and P/FP*∗*10 at all levels of PEEP at all the three time points is shown in Figure 2.

  P/FP*∗*10 (B) = (PaO_2_*∗*10)/(FiO_2_*∗*PEEP) at baseline (the value obtained at one hour after intubation and initiation of MV); S/FP*∗*10 (B) = (SpO_2_*∗*10)/(FiO_2_*∗*PEEP) at baseline (the corresponding values obtained at one hour after intubation and MV).
  P/FP*∗*10 (D1) = (PaO_2_*∗*10)/(FiO_2_*∗*PEEP) at day 1 (worst values of P/FP*∗*10 within 1–24 hours of intubation); S/FP*∗*10 (B) = (SpO_2_*∗*10)/(FiO_2_*∗*PEEP) at day 1 (the corresponding values of S/FP*∗*10 within 1–24 hours of intubation).
  P/FP*∗*10 (D3) = (PaO_2_*∗*10)/(FiO_2_*∗*PEEP) at day 3 (worst values of P/FP*∗*10 within 48–72 hours of intubation); S/FP*∗*10 (D3) = (SpO_2_*∗*10)/(FiO_2_*∗*PEEP) at day 3 (the corresponding values of S/FP*∗*10 within 48–72 hours of intubation). PEEP: positive end-expiratory pressure.

Among all the P/FP*∗*10 and S/FP*∗*10 ratios at baseline, day one, and day three and the changes in the ratios from baseline and day one to day three of MV, the change in P/FP*∗*10 (D3-B) has the best ability to predict survival in ARDS patients, with AUC of 0.902 and a cut-off ≥34.25 (Table 3). Among the S/FP*∗*10 ratios, the S/FP*∗*10 (D3-B) has the highest AUC and specificity to predict survival (AUC: 0.877, *p* < 0.001, sensitivity: 73.5%, specificity: 97%, CI: 0.803–0.952, cut-off ≥64.40). The AUC values of the P/FP*∗*10 (B), P/FP*∗*10 (D1), P/FP*∗*10 (D3), P/FP (D3-B), P/FP (D3-D1), S/FP*∗*10 (B), S/FP*∗*10 (D1), S/FP*∗*10 (D3), S/FP (D3-B), and S/FP (D3-D1) with their respective cut-off values are depicted in Table 3.

**Figure 3:**
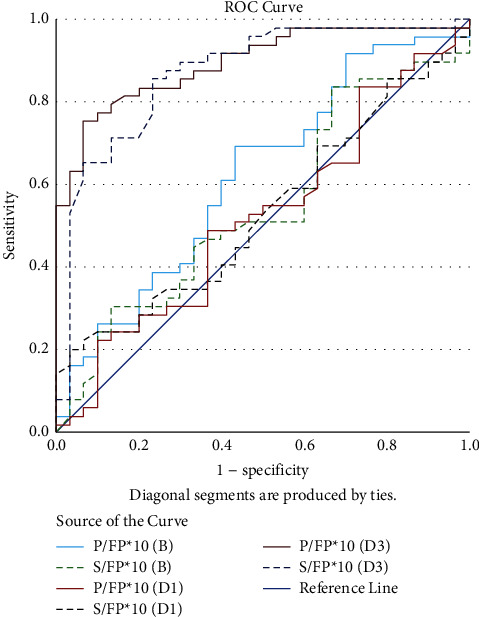
Receiver operating characteristic curves to predict survival for P/FP*∗*10 and S/FP*∗*10 at baseline, day 1, and day 3. The P/FP*∗*10 (D3) has an AUC of 0.900 and a cut-off ≥257.5 to predict survival, followed by S/FP*∗*10 (D3) with a cut-off ≥243.75 and an AUC of 0.872.

The ROC curves of the P/FP*∗*10 (B), P/FP*∗*10 (D1), P/FP*∗*10 (D3), S/FP*∗*10 (B), S/FP*∗*10 (D1), and S/FP*∗*10 (D3) to predict survival are depicted in Figure 3.

In Figure 3, PaO_2_ = partial pressure of arterial oxygen, SpO_2_ = pulse oximetry oxygen saturation, FiO_2_ = fraction of inspired oxygen, PEEP = positive end-expiratory pressure, P/FP*∗*10 (B)= (PaO_2_*∗*10)/(FiO_2_*∗*PEEP) (the value obtained at one hour after intubation and initiation of mechanical ventilation), S/FP*∗*10 (B) = (SpO_2_*∗*10)/(FiO_2_*∗*PEEP) (the corresponding values obtained at one hour after intubation and mechanical ventilation), P/FP*∗*10 (D1) = worst values of P/FP*∗*10 day 1 (within 1–24 hours of intubation), S/FP*∗*10 (D1) = corresponding values of S/FP*∗*10 on day 1, P/FP*∗*10 (D3) = worst values of P/FP*∗*10 day 3 (within 48–72 hours of intubation), and S/FP*∗*10 (D3) = corresponding values of S/FP*∗*10 on day 3 (within 48–72 hours of intubation.

**Figure 4:**
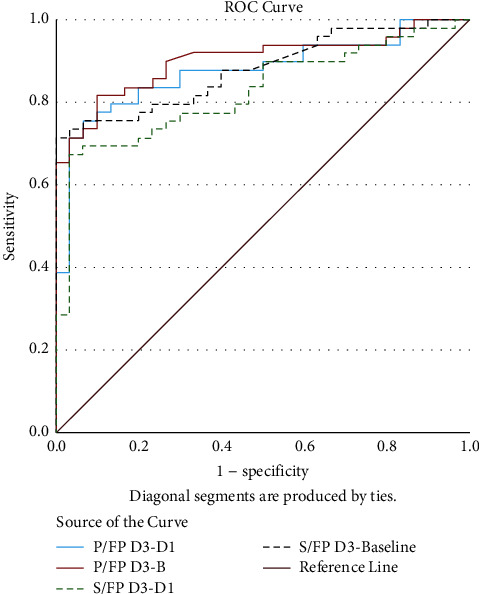
The ROC of the change in the P/FP*∗*10 and S/FP*∗*10 ratios from baseline and day one to day three to predict survival. The AUC of the P/FP (D3-B) is the highest (AUC: 0.902, cut-off ≥34.25), followed by the AUC of S/FP (D3-B) (AUC: 0.877, cut-off ≥64.40).

The ROC of the changes in oxygenation as depicted by P/FP (D3-B), P/FP (D3-D1), S/FP (D3-B), and S/FP (D3-D1) to predict survival is displayed in Figure 4.

In Figure 4, S/FP (D3–B) change in S/FP*∗*10 from baseline to day three, S/FP (D3–D1) change in S/FP*∗*10 from day one to day three, P/FP (D3-B) change in P/FP*∗*10 from baseline to day three, P/FP (D3-D1) change in S/FP*∗*10 from day one to day three. AUC = area under curve.

**Figure 5:**
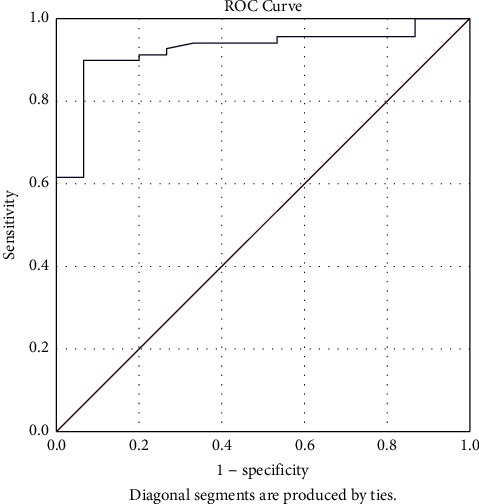
ROC curve depicting the discriminant function of S/FP*∗*10 (B) to be categorized as severe ARDS as per Berlin definition (AUC: 0.925, *p* < 0.001, cut-off value ≤116, CI: [0.862–0.988]). S/FP*∗*10 (B) = (SpO_2_*∗*10)/(FiO_2_*∗*PEEP) (the values obtained at one hour after intubation and mechanical ventilation).

The S/FP*∗*10 (B) has excellent discrimination ability to predict severe ARDS category and revealed an AUC of 0.925, *p* < 0.001, 90% sensitivity, 93% specificity, cut-off value ≤116, and CI of [0.862–0.988]. The ROC of the S/FP*∗*10 (B) discriminant function to predict severe ARDS is depicted in Figure 5.

**Figure 6:**
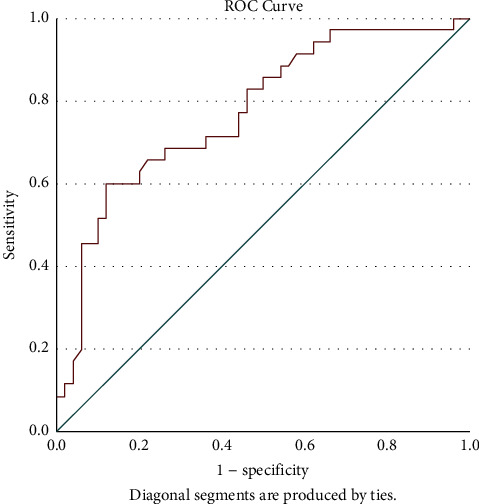
ROC curve depicting the discriminant function of S/FP*∗*10 (B) to be categorized as mild ARDS as per Berlin definition (AUC: 0.773, *p* < 0.001, cut-off value ≥190, sensitivity: 69%, specificity: 74%, 95% CI: [0.672–0.875]. S/FP*∗*10 (B) = (SpO_2_*∗*10)/(FiO_2_*∗*PEEP) (the values obtained at one hour after intubation and mechanical ventilation).

S/FP (B) also had fair discrimination ability to predict mild ARDS category and revealed an AUC of 0.773, *p* < 0.001, sensitivity of 69%, specificity of 74%, and 95% CI of [0.672–0.875] with a cut-off value ≥190. The ROC of the S/FP*∗*10 (B) discriminant function to predict mild ARDS is depicted in Figure 6.

Thus, as per our results, the moderate ARDS category will be S/FP*∗*10 (B) ≥116 and ≤190.

In order to compare the reliability of S/FP to predict outcomes as compared to P/FP, the comparison of the ROC curves was done using the DeLong test using MedCalc software version 20.111. The ROC of the S/FP*∗*10 to P/FP*∗*10 at three time points (baseline, day one, and day three) to predict outcome in terms of survival was calculated. It showed that the null hypothesis was acceptable, and there was no statistically significant difference in the AUC between the ROC curves of S/FP*∗*10 (B) and P/FP*∗*10 (B), S/FP*∗*10 (D1) and P/FP*∗*10 (D1), and S/FP*∗*10 (D3) to P/FP*∗*10 (D3) for survival outcome prediction (*p*=0.054, *p*=0.681, and *p*=0.264, respectively). Similarly, there was no difference in the AUC of the ROC curves between S/FP (D3-D1) and P/FP (D3-D1) as well as S/FP (D3-B) and P/FP (D3-B) to predict survival (*p*=0.087and 0.463, respectively).

## 6. Discussion

A recent study has concluded that in ARDS patients, when PEEP is integrated into the P/F ratio, the validity to predict hospital mortality is amplified [2]. The authors stated that the incorporation of PEEP into the P/F ratio enables the reflection of lung compliance and lung recruitment during the evaluation of the severity of ARDS [2]. The setting of PEEP in most cases of severe ARDS has been said to be approximately 8 cm H_2_O, which is low [2, 7]. In the above-mentioned study, the AUC for prediction of mortality in cases of PEEP of about 8 cm H_2_O was higher for P/FP*∗*10 (0.775) as compared to AUC of 0.691 for P/F ratio [2]. The sensitivity to predict mortality was also higher for P/FP*∗*10 (84.3%) compared to 65% for P/F [2]. The authors of the study included a correction factor of 10 for the P/FP as a PEEP of about 10 cm H_2_O is commonly set as the initial ventilator setting [2, 8]. The other reason why factor 10 was chosen was that a regression line plotted in the study between P/F ratio and PEEP intersected the P/F of value 150 mmHg (which usually discriminates the survivors and non-survivors) at a PEEP of 10 cm H_2_O [2]. We used the same factor (10) to be multiplied by the S/FP ratio as was done in the previous study [2]. For our study, we wanted to use a noninvasive, easily plausible, and continuous oxygen monitoring method such as SpO_2_ after incorporating the PEEP into S/F ratio for correlation to the P/FP*∗*10. The P/FP*∗*10 ratio has now proven to be more pertinent as compared to the P/F ratio for prognostication of outcomes in ARDS patients [2]. Thus, we evaluated the correlation of S/FP*∗*10 to P/FP*∗*10 in ARDS patients at baseline, day one, and day three of MV. The reason for selecting the baseline and the worst values of S/FP*∗*10 up to day one of MV for correlation to P/FP*∗*10 was based on previous ARDS studies that demonstrated that compared to baseline oxygenation, values at 24 hours are more significant to predict outcomes [9–11]. Another study concluded that 6–12 hours of ARDS onset accurately predicted outcomes and was the optimal time for recruitment of patients into clinical research [12]. Thus, we selected the time interval between one hour and 24 hours of MV as day one. After initiation of MV, an ABG done within one hour has been proven to improve mechanical ventilation intervention and monitoring, by significantly detecting respiratory acidosis [13]. Thus, we chose the S/FP*∗*10 and P/FP*∗*10 values within one hour of MV initiation as baseline for correlation analysis. The strength of correlation between S/FP*∗*10 and P/FP*∗*10 in our study was more than the correlation between P/F and S/F at the respective time intervals. A study comparing the correlation between only SpO_2_ and PaO_2_ was done previously, which showed that the Pearson correlation coefficient was 0.423 only [14]. If FiO_2_ is incorporated in the correlation, the strength of the correlation improves to about 0.580–0.766 as we found in our study. However, if PEEP is incorporated, the strength of correlation increases and is highly significant (0.733–0.853) as we found in our study. Moreover, in patients with PEEP ≥10 cm H_2_O, there is a good correlation of S/FP*∗*10 and P/FP*∗*10, which is excellent on day three (0.881). This is clinically very significant because it is in this group of ARDS patients who require a higher PEEP that frequent ABG is ordered to evaluate the P/F ratios. Often as the days of MV progress from day one to day three, the need for placing an invasive arterial catheter for repeated ABG is often contemplated upon. The findings of the excellent correlation between S/FP*∗*10 and P/FP*∗*10 on day three in our study shows that the need for invasive arterial catheter placement for the purpose of frequent ABG monitoring as days of MV progress, can be avoided. Even though previous literature shows a good correlation between S/F ratio and P/F ratio, PEEP component which is so crucial was not incorporated previously [15].

A recent study on 1034 ARDS patients concluded that mortality prediction from oxygenation could be more conclusively done on day three (after 48 hours) of ARDS onset [16]. As the most common cause of mortality in ARDS is multi-organ dysfunction, which evolves and progresses over time, oxygenation values on the third day (after 48 hours) are likely to have a better predictive value for mortality [16–18]. Therefore, we selected the P/FP*∗*10 and S/FP*∗*10 values at day three (48–72 hours after MV), apart from baseline (0-1 hour after MV) and day one (1–24 hours after MV), to predict survival. The S/F ratio complements the P/F ratio; thus, S/F has also been incorporated to reflect respiratory status in the modified Sequential Organ Failure Assessment (mSOFA) score [19]. Similar to the manner in which a change of SOFA score during the first 48 hours of ICU stay has been shown to predict mortality, we also hypothesized that change of S/FP*∗*10 from initiation of MV to day three of MV may predict survival in a more clinically significant and validated manner [20]. We found that as compared to the P/FP*∗*10 and S/FP*∗*10 values at baseline and day one, the values on day three were more significant for predicting survival. Both the change in P/FP*∗*10 and the change in S/FP*∗*10 values between the baseline and day three values had one of the highest AUCs to predict survival. Previous research has shown that SpO_2_/FiO_2_ classification (≥190 or <190) along with PEEP categorization (≥10 cm H_2_O or <10 cm H_2_O) was useful for predicting mortality in moderate-severe ARDS at 24 hours of ARDS onset*p*=0.087 [21]. However, the results of our study were different as compared to the previous research, and we did not find S/FP*∗*10 (D1) to be a predictor of survival in ARDS patients. This difference in finding could be due to the fact that, in our study, we took the worst values of P/F within 1–24 hours after initiation of MV, and then the corresponding value of S/FP*∗*10 (D1) was calculated. Thus, the worst value of P/F and the corresponding S/FP*∗*(10) within 1–24 hours of MV could have been even after just few hours of the baseline time point and not exactly at 24 hours of MV initiation. Thus, the S/FP*∗*10 (D1) was probably not able to predict outcomes, as it may not exactly represent the 24-hour time point values, and could have represented values much closer to baseline. However, the S/FP*∗*10 (D3), S/FP*∗*10 (D3-B), and S/FP*∗*10 (D3-D1) were able to reliably predict outcomes in ARDS patients, showing that as compared to the baseline values, the improvement or deterioration in S/FP*∗*10 values over a certain time period is more helpful in predicting outcomes. This finding was in agreement with those of a recent study on ARDS patients [16].

In a previous study on P/FP*∗*10, Palanidurai et al. studied its utility for predicting mortality in ARDS only on the day of intubation [2]. However, the authors concluded in their hypothesis that the predictive ability of P/FP*∗*10 to predict mortality will improve over 24–72 hours [2]. This is exactly what we found in our study. Not only did the P/FP*∗*10 have an excellent predictive value for survival at 48–72 hours, but S/FP*∗*10 also had a good predictive ability for survival at 48–72 hours, as compared to baseline and day one.

Thus, clinicians may use the S/FP*∗*10 parameters in a mechanically ventilated ARDS patient, rather than repeated ABGs to predict survival. However, the S/FP*∗*10 values at baseline or on day one were not able to predict survival. Our findings were like the results of the trial by Chiu et al., where the authors concluded that the change in oxygenation status over 48 hours of ARDS onset, rather than baseline values, is significant [16]. The change in oxygenation as depicted by S/FP*∗*10 change in our study being an important predictor of survival is also theoretically validated by previous studies [22, 23]. In situations when a patient's oxygenation improves after prone position ventilation, the change in oxygenation will be significant in 48 hours after a single proning session [22]. Such patients are more likely to survive as compared to those in whom there is no significant improvement in oxygenation over a period of MV.

In a study done by Fukuda et al. on hypoxemic respiratory failure patients who had bilateral lung field opacities, the AUC of S/F ratio at 24 hours of ICU admission to predict mortality of ICU stay was 0.784 [24]. However, we found that, rather than the P/FP*∗*10 and S/FP*∗*10 at baseline or at day one, it is the change in P/FP*∗*10 and S/FP*∗*10 from baseline to day three of MV that has the highest AUC to predict survival (AUC of 0.902 and 0.877, respectively). In our study, the S/FP (D3-B) ≥64 and S/FP (D3-D1) ≥75 have good validity for predicting survival with AUC of 0.877 and 0.821, respectively. Other than PEEP, indices of ventilator parameters such as mean airway pressure and plateau pressure have also been incorporated into P/F ratio for better predictor of outcomes, but these require paralyzing the patient for accurate measurement [2]. Thus, incorporation of PEEP into S/F ratio and assessing the change may be a practical yet reliable predictor of outcome in ARDS patients.

Apart from the correlation to the P/FP ratio and prediction of survival, we felt that another utility of S/FP*∗*10 that should be examined is its discriminating ability to predict severe ARDS category as per Berlin criteria. The discrimination ability should also be examined early, after intubation (baseline), so that clinicians may plan interventions in severe ARDS patients accordingly. We found that the S/FP*∗*10 (B) ≤116 has excellent discrimination ability to predict severe ARDS category with an AUC of 0.925 and 90% sensitivity along with 93% specificity. This was much higher than the AUC of 0.839 for S/F to predict P/F >200 as per recently concluded study results [25]. However, the study included hypoxemic patients, and not patients with ARDS [25].

We evaluated the ability of S/FP*∗*10 (B) to predict severe ARDS, as these patients require early assiduous treatment planning and interventions such as lung-protective ventilation, prone ventilation, neuromuscular blockade, and a targeted negative fluid balance and are at greater risk of multiple organ dysfunction [26].

The correlation between S/FP*∗*10 and P/FP*∗*10 at baseline (*r* 0.831), day one (*r* 0.733), and day three (*r* 0.853) in our study was much higher compared to the correlation between S/F ratio and P/F ratio in the above-mentioned study (*r* 0.66). Thus, the consideration of PEEP applied strengthens the correlation between S/FP*∗*10 and P/FP*∗*10 ratios.

In the study done by Pandharipande et al., the authors concluded that the correlation of S/F and P/F was unaffected by the various levels of PEEP (<8 cm H_2_O, 8–12 cm H_2_O, and >12 cm H_2_O) [27]. The results of our study were similar in the aspect that we found that, even in the subgroup of patients with higher PEEP ≥10 cm H_2_O, the correlation of P/F and S/F and P/FP and S/FP was similar to that of the entire group of patients with various PEEP levels. Furthermore, in the above-mentioned study, when the contribution of PEEP was accounted for, the correlation between S/F and P/F was stronger as compared to the correlation between S/F and P/F without contribution of PEEP, as we found in our study [27].

The study's strengths are that it has incorporated the concept of PEEP into a noninvasive method of oxygenation assessment (S/FP*∗*10) in ARDS patients to predict survival. The need for doing repeated ABGs may be avoided. This is important as, apart from the risk of infection and costs, arterial cannulation may even lead to devastating complications such as symmetrical peripheral gangrene [28]. The excellent correlation of S/FP*∗*10 to P/FP*∗*10 at higher PEEP on day three may help avoid arterial cannulation for repeated P/F or P/FP assessment in patients with PEEP ≥10 cm H_2_O, as the days of MV progress in ICU. Similar to the manner in which a change in SOFA scores predicts mortality, the study could validate that the change in P/FP*∗*10 as well as S/FP*∗*10 ratio over 48 hours after intubation is a reliable predictor of survival. The study could also conclude that S/FP*∗*10 is able to predict severe ARDS category patients early after intubation, with an excellent discriminant function.

The study has certain limitations. It was a single-centre study. In addition, we did not plot the ROC of S/FP*∗*10 to predict survival at different levels of PEEP as done by Palanidurai et al. in their study on P/FP*∗*10 [2]. We assessed the discriminant function of S/FP*∗*10 to predict only severe ARDS, and not mild or moderate ARDS. The limitations of pulse oximetry use like poor waveform, hypothermia, shock, and methemoglobinemia decreases the utility of S/FP ratio as compared to P/FP ratio [25].

## 7. Conclusions

S/FP*∗*10 has a strong correlation to P/FP*∗*10 in ARDS patients at baseline, day one, and day three of MV, which is stronger than the correlation between S/F and P/F ratios at the respective time intervals. S/FP*∗*10 (B) ≤116 has an excellent discriminant function to predict severe ARDS category. On day three after initiation of MV, S/FP*∗*10 ≥244 has a good predictive ability for survival, whereas an increase in S/FP (D3-B) by ≥64 and increase in S/FP (D3-D1) by ≥75 also have good predictive ability for survival.

## Figures and Tables

**Table 1:** Depiction of the mean and standard deviation of the demographic and baseline variables in 85 patients.

Variables	*N*	Mean ± SD
Age in years	85	54 ± 15
Gender	Males = 60 (71%)	
Survivors	48 (57%)	
APACHE II score	85	18.66 ± 7.02
SOFA score	85	8.24 ± 3.62
Mechanical ventilation days	85	6.17 ± 6.41
Length of ICU stay	85	7.49 ± 6.65
FiO_2_ (B)	85	0.7 ± 0.21
PEEP (B), cm H_2_O	85	8.94 ± 2.66
SpO2 (B)	85	94.11 ± 5.36
P/F (B) ratio	85	178.77 ± 63.36
P/FP*∗*10 (B) ratio	85	225.94 ± 123.54
S/F (B) ratio	85	148.10 ± 51.82
S/FP*∗*10 (B) ratio	85	189.85 ± 109.47
Etiology of ARDS	Sepsis, 25	
	Pneumonia, 24	
	Acute febrile illness, 23	
	Pancreatitis, 7	
	Others (poisoning, snake bite), 6	

APACHE: acute physiology and chronic health evaluation; SOFA: Sequential Organ Failure Assessment; FiO_2_ (B): fraction of inspired oxygen at baseline; PEEP (B): positive end-expiratory pressure at baseline; PaO_2_: partial pressure oxygen in arterial blood; SpO_2_ (B): pulse oximetry oxygen saturation at baseline; P/F (B) ratio: PaO_2_/FiO_2_, partial pressure of arterial oxygen to inspiratory oxygen fraction ratio; P/FP*∗*10 (B): PaO_2_*∗*10/FiO_2_*∗*PEEP at baseline; S/FP*∗*10 (B) ratio: SpO_2_*∗*10/FiO_2_*∗*PEEP; ARDS: acute respiratory distress syndrome.

**Table 2:** Correlation between P/FP*∗*10 and S/FP*∗*10 at various time points and at high PEEP.

Time points	Pearson correlation coefficient (*r*) between P/F and S/F	*p* value	Pearson correlation coefficient (*r*) between P/FP*∗*10 and S/FP*∗*10	*p* value
*All enrolled patients*				
Baseline (*n* = 85)	0.660	<0.001	**0.831**	<0.001
Day 1 (*n* = 85)	0.580	<0.001	**0.733**	<0.001
Day 3 (*n* = 79)	0.766	<0.001	**0.853**	<0.001

*Values with PEEP ≥10 cm H*_*2*_ *O*				
Baseline (*n* = 39)	0.634	<0.001	**0.687**	<0.001
Day 1 (*n* = 26)	0.625	0.001	**0.726**	<0.001
Day 3 (*n* = 31)	0.851	<0.001	**0.881**	<0.001

**Table 3:** Depiction of the predictive ability for survival for the various P/FP*∗*10 and S/FP*∗*10 ratios at different time intervals.

Variables	Cut-off value	AUC	*p* value	Sensitivity (%)	Specificity (%)	95% CI
S/FP*∗*10 (B)	≥162	0.558	0.391	52	53	0.427–0.689
P/FP*∗*10 (B)	≥187.6	0.635	**0.046**	69	58	0.507–0.763
P/FP*∗*10 (D1)	176.8	0.534	0.618	55	53	0.401–0.666
S/FP*∗*10 (D1)	≥157.5	0.545	0.508	53	55	0.416–0.674
P/FP*∗*10 (D3)	≥257.5	0.900	**<0.001**	80	87	0.833–0.968
S/FP*∗*10 (D3)	≥243.75	0.872	**<0.001**	78	77	0.787–0.956
S/FP (D3-B)	≥**64.40**	**0.877**	**<0.001**	**73.5**	**97**	0.803–0.952
S/FP (D3-D1)	≥75.62	0.821	**<0.001**	71.4	80	0.728–0.914
P/FP (D3-B)	≥**34.25**	**0.902**	**<0.001**	81.6	90	0.834–0.970
P/FP (D3-D1)	≥28.56	0.876	**<0.001**	83.7	80	0.798–0.953

PaO_2_ = partial pressure of arterial oxygen, SpO_2_ = pulse oximetry oxygen saturation, FiO_2_ = fraction of inspired oxygen, PEEP = positive end-expiratory pressure, P/FP*∗*10 = (PaO_2_*∗*10)/(FiO_2_*∗*PEEP), S/FP*∗*10 = (SpO_2_*∗*10)/(FiO_2_*∗*PEEP), P/FP*∗*10 (B) = the value obtained at one hour after intubation and initiation of MV, S/FP*∗*10 (B) = corresponding values obtained at one hour after intubation and MV, P/FP*∗*10 (D1) = worst values of P/FP*∗*10 day 1 (within 1–24 hours of intubation), S/FP*∗*10 (D1) = corresponding values of S/FP*∗*10 on day 1, P/FP*∗*10 (D3) = worst values of P/FP*∗*10 day 3 (within 48–72 hours of intubation), S/FP*∗*10 (D3) = corresponding values of S/FP*∗*10 on day 3 (within 48–72 hours of intubation), S/FP (D3-B) = change in S/FP*∗*10 from baseline to day three, S/FP (D3-D1) = change in S/FP*∗*10 from day one to day three, P/FP (D3-B) = change in P/FP*∗*10 from baseline to day three, and P/FP (D3-D1) = change in S/FP*∗*10 from day one to day three.

## Data Availability

The data sets used and analyzed during the current study are available from the first and corresponding author upon reasonable request. The data are not publicly available due to them containing information that could compromise research participant privacy/consent.

## Abbreviations

ARDS: Acute respiratory distress syndrome
PaO_2_: Partial pressure of arterial oxygen
FiO_2_: Fraction of inspired oxygen
PEEP: Positive end-expiratory pressure
P/F ratio: PaO_2_/FiO_2_, partial pressure of arterial oxygen to inspiratory oxygen fraction ratio
P/FP*∗*10: PaO_2_*∗*10/FiO_2_*∗*PEEP
SpO_2_: Pulse oximetric oxygen saturation
S/F ratio: SpO2/FiO2, partial pressure of arterial oxygen to inspiratory oxygen fraction ratio
S/FP*∗*10: SpO_2_*∗*10/FiO_2_*∗*PEEP
ICU LOS: Length of stay in intensive care unit
MV: Mechanical ventilation
CI: Confidence interval
APACHE II: Acute physiology and chronic health evaluation II
SOFA score: Sequential Organ Failure Assessment score.
